# Novel Technique for Laparoscopic Common Bile Duct Exploration Using Endovascular Instrumentation

**DOI:** 10.7759/cureus.4041

**Published:** 2019-02-09

**Authors:** Daniel Copeland, Elizabeth E Blears, Zhihao Zhu, Anthony Nguyen, Russell Van Husen

**Affiliations:** 1 Surgery, Midland Surgical Associates, Midland, USA; 2 Surgery, University of Texas Medical Branch, Galveston, USA; 3 Miscellaneous, University of Texas Medical Branch, Galveston, USA; 4 Neurosurgery, University of Texas Medical Branch, Galveston, USA

**Keywords:** laparoscopic cholecystectomy, acute cholecystitis, choledocholithiasis, laparoscopic surgery, minimally invasive surgery, laparoscopy, endovascular surgery, surgical technique

## Abstract

Treatment of choledocholithiasis is sometimes a two-step process involving both surgeons and gastroenterologists. Common bile duct (CBD) exploration can be performed at the same time as cholecystectomy but often requires the use of rigid tools, increasing the risk of CBD damage. Here, we report the case of a 64-year-old man who presented with epigastric pain and a positive Murphy's sign. Ultrasonography revealed cholecystitis with cholelithiasis. Gangrenous cholecystitis was visualized upon surgical exploration, and an intraoperative cholangiogram diagnosed likely choledocholithiasis. Cholecystectomy was completed, and CBD exploration was performed by the manipulation of endovascular equipment using a trans-cystic approach through to the ampulla of Vater, and the patient made a complete recovery without complications. The substantial flexibility, gentleness, and durability of endovascular instruments allow for minimal tension on structures during the removal of gallstones from the CBD, providing safe, definitive treatment for choledocholithiasis during cholecystectomy.

## Introduction

Cholelithiasis affects an estimated 10%-15% of adults in the United States [1]. Choledocholithiasis is a relatively common complication of cholelithiasis, occurring in approximately 20% of patients with gallstones [2]. Although recognizable by some preoperative scans, choledocholithiasis can remain occult until intraoperative cholangiogram (IOC) is performed. However, the recognition of choledocholithiasis is important, as it is associated with cholangitis and obstructive pancreatitis, increasing the risk of death nine-fold as compared to patients with uncomplicated gallstones [2-3]. The management of patients with suspected choledocholithiasis has been controversial, involving a variety of open surgical, minimally invasive surgical (such as laparoscopic and robotic), endoscopic, and ultrasonic modalities [4-5]. While open or minimally invasive surgical techniques provide an effective treatment of choledocholithiasis at the same time as cholecystectomy, they often involve choledochotomy and instrumentation with inflexible laparoscopic tools, such as retrieval baskets, which can cause damage to the biliary tree if too much force is applied. These invasive maneuvers are suspected to result in higher rates of complications such as stricture [6], bile leakage [7], higher conversion rate to open cholecystectomy as compared to laparoscopic cholecystectomy with endoscopic retrograde cholangiopancreatography (ERCP) [8].

While ERCP does not involve this manipulation of the biliary tree and thus avoids the increased risk of conversion to an open procedure, it is associated with an approximately 11.5-minute longer total operative time [8]. However, in medically higher-risk patients, ERCP versus laparoscopic cholecystectomy with common bile duct exploration is associated with a lower rate of duct clearance and a higher number of procedures per patient [9]. To optimize the benefits of a one-time, intraoperative treatment for choledocholithiasis while minimizing the risks to the biliary tree during stone removal, we discuss a novel, minimally invasive technique for common bile duct exploration. This technique helps provide therapeutic intervention at the time of laparoscopic cholecystectomy, avoiding the need for post-operative ERCP. It also uses a trans-cystic (via the cystic duct) approach rather than a trans-ductal approach (via an additional choledochotomy in the common bile duct), which provides a less invasive method of common bile duct intervention. Moreover, when typical common bile duct exploration is performed via the trans-cystic route, it often uses laparoscopic instruments that are often bulky, rigid, or articulated in fixed positions so that there is much tension placed on the biliary tree during manipulation, leading to therapeutic failure in cases where the cystic duct and hepatic duct meet at a small angle [10]. In our approach, endovascular instruments originally designed for the clearance of plaque from blood vessels are introduced via the trans-cystic route and are extremely flexible, yet durable, to provide treatment of the biliary tree without significant tension. Thus, we discuss a case of a 64-year-old male who presented with acute gangrenous cholecystitis secondary to cholelithiasis who was successfully managed with this technique.

## Case presentation

A 64-year-old male presented to the emergency department with acute onset epigastric pain. Work-up with admission ultrasound revealed a common bile duct of 7.7 mm, pericholecystic fluid, positive sonographic Murphy’s sign, and cholelithiasis. The patient had a past medical history of hepatitis C secondary to intravenous drug abuse, alcohol abuse, major depressive disorder, and insomnia. He was afebrile throughout his hospital course without tachycardia or hypotension. He did not have any abnormal elevations in his total bilirubin or liver function tests at admission. At admission, his white blood cell (WBC) count was 12.07 x 103 /uL with 87% neutrophils. He was taken to surgery on the day of admission for laparoscopic cholecystectomy with IOC.

**Figure 1:**
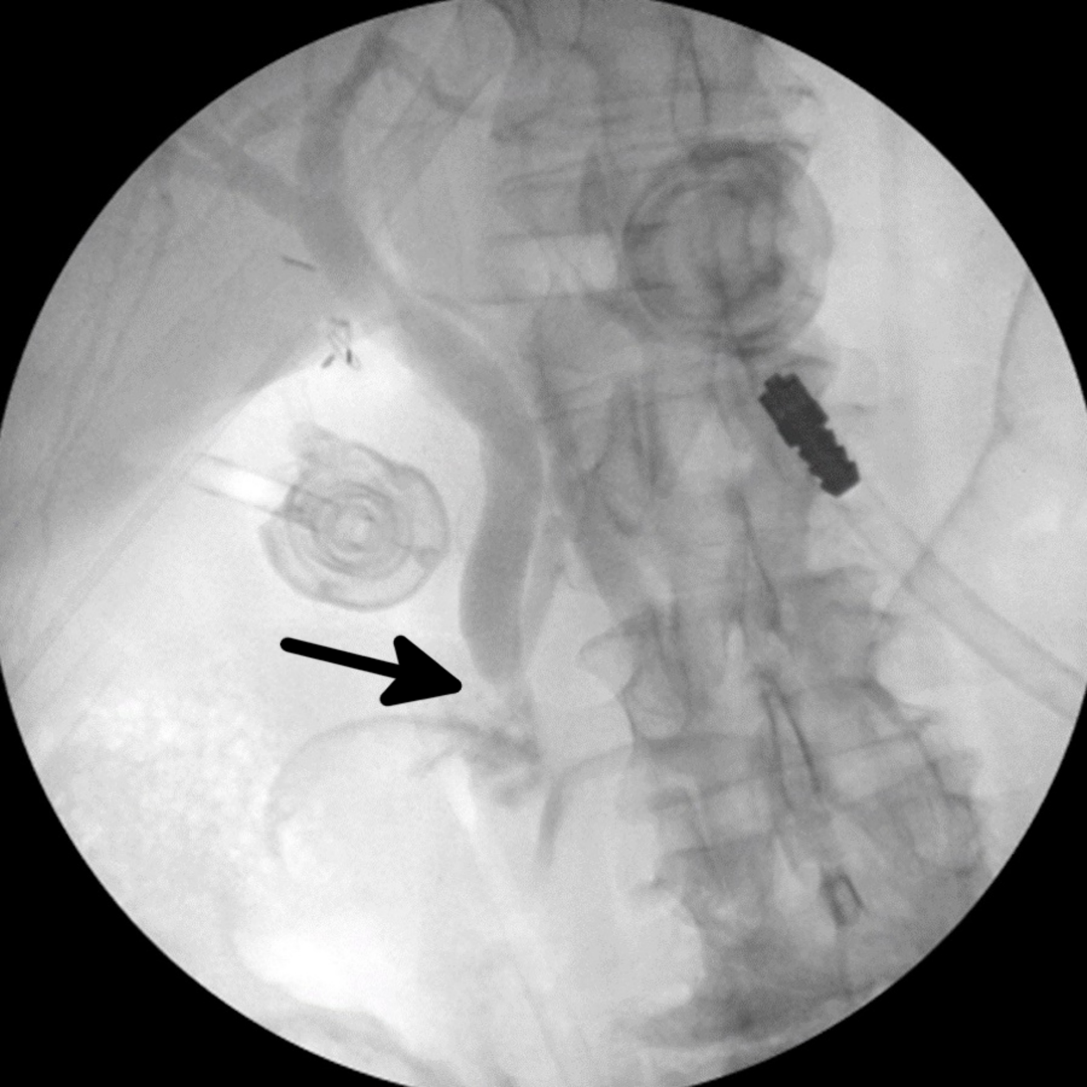
Intraoperative cholangiogram prior to common bile duct exploration demonstrating obstruction of flow near the ampulla of Vater (black arrow). This is suggestive of choledocholithiasis.

Upon gross inspection of the intraperitoneal cavity, pus surrounded aspects of the end of the liver capsule and the gallbladder appeared gangrenous. A large stone was palpable with laparoscopic instruments within the infundibulum of the gallbladder. After dissecting the cystic duct free of surrounding inflamed tissue, a partial transection of the cystic duct was made so that a cholangiogram catheter could be threaded into the cystic duct and clipped to secure the catheter. A cholangiogram was subsequently performed with a taut radiopaque introducer (Teleflex Medical, Wayne, Pennsylvania, US) and needle (3.0mm x 2.4mm x 8.9 cm) and a 4.5 Fr (1.5mm) x 45.7 cm taut operative cholangiogram catheter (Teleflex Medical). The initial image of the cholangiogram is shown in Figure 1, demonstrating a lack of contrast in the common bile duct near the ampulla of Vater, suggestive of choledocholithiasis.

**Figure 2:**
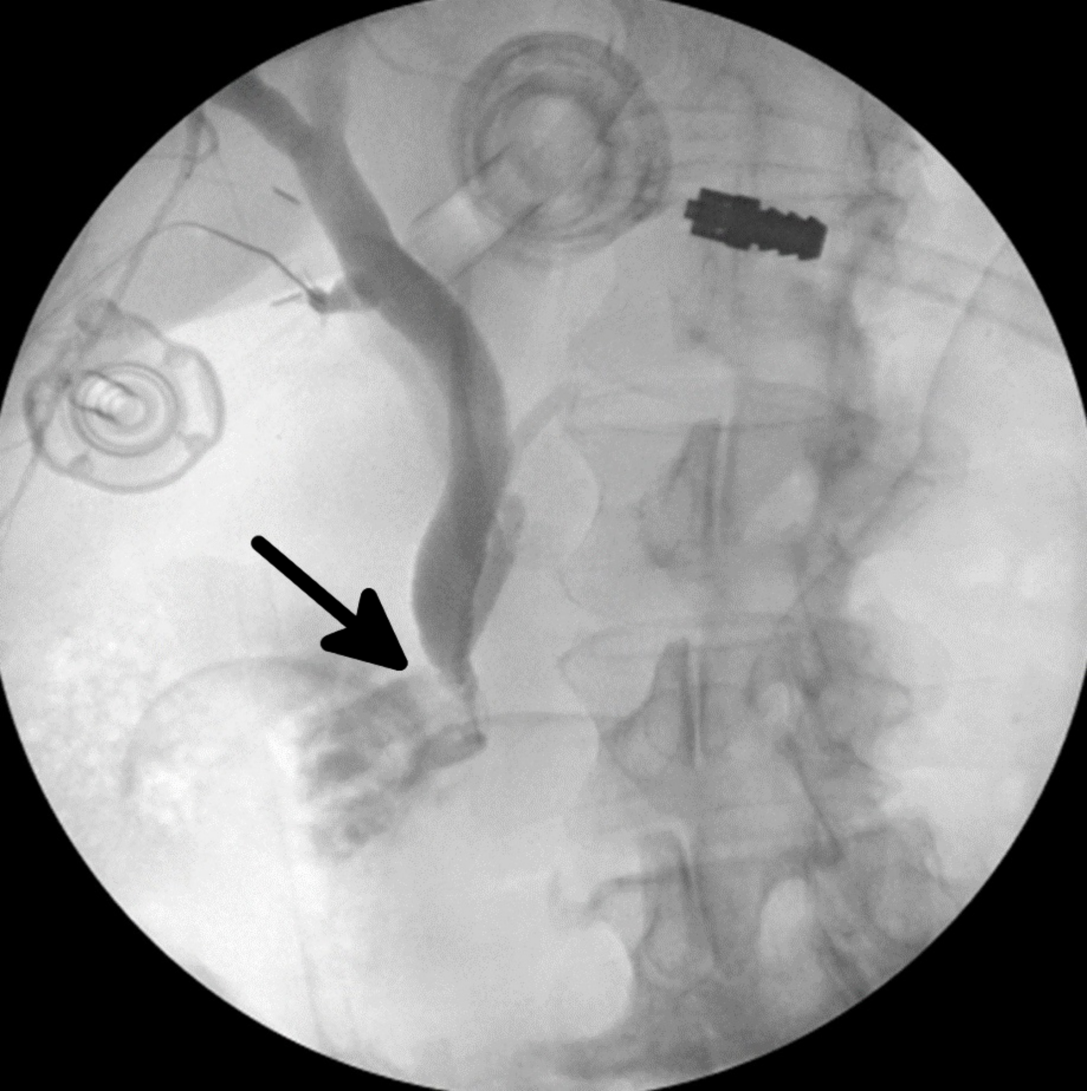
Intraoperative cholangiogram following glucagon administration. In comparison to the previous cholangiogram, this one demonstrates little improvement in flow past the ampulla of Vater (black arrow), suggesting persistence of choledocholithiasis.

**Figure 3:**
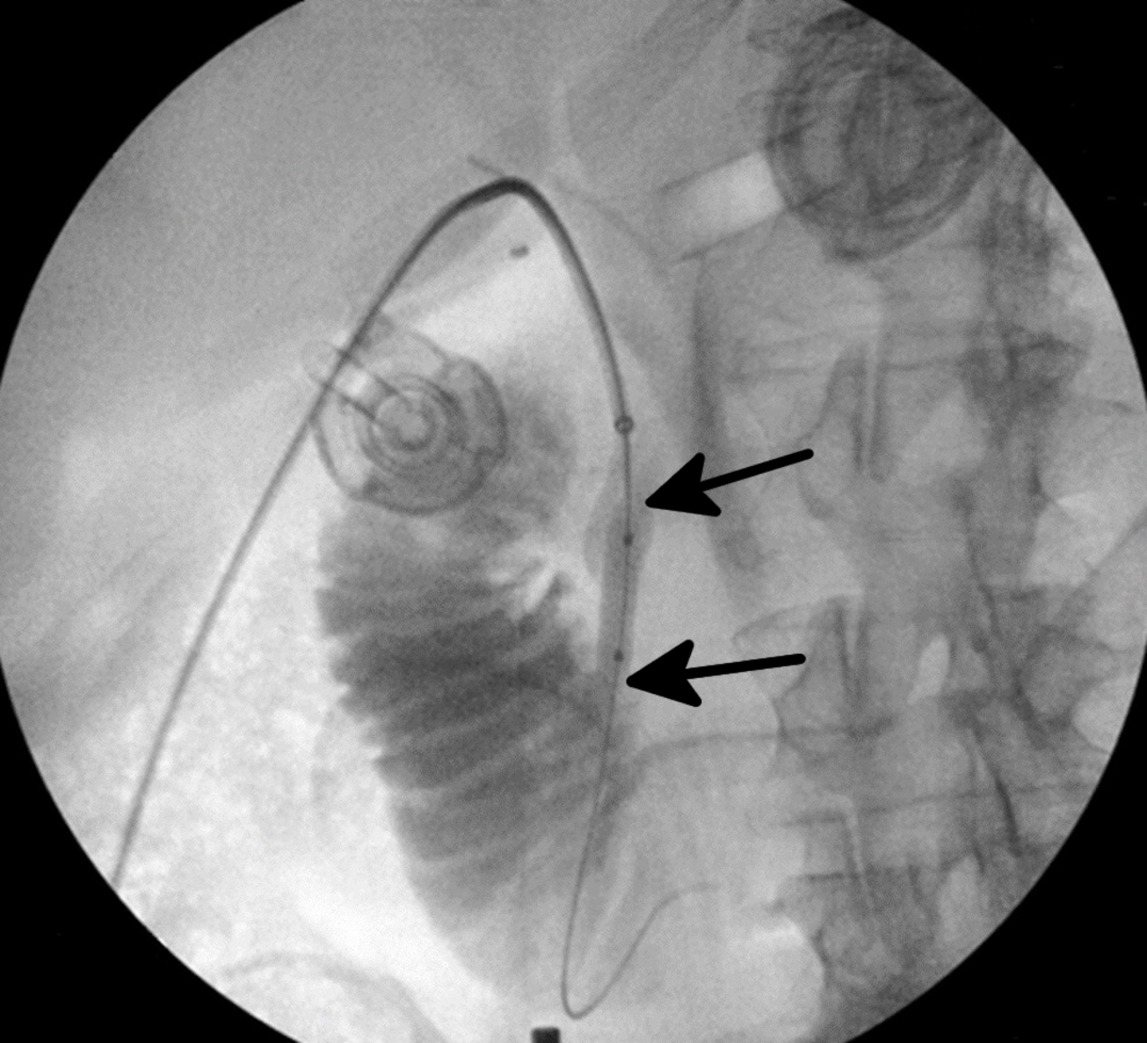
Radiographic imaging demonstrating a glidewire and TEMPO AQUA® catheter passed through the common bile duct and ampulla of Vater (black arrows). TEMPO AQUA® (Cordis, Milpitas, California, US)

Opacification within the area of the ampulla as well as the reflux of contrast medium into the pancreatic duct suggested the presence of a stone in the proximal common bile duct. One gram of glucagon was administered to the patient and two minutes later, an IOC was repeated. This second cholangiogram showed persistent opacification at the ampulla and limited contrast medium reaching the duodenum, with reflux into the pancreatic duct (Figure 2). Rather than performing a conventional choledochotomy on the common bile duct and exploring the common bile duct with a choledochoscope, an endovascular equipment set was employed to provide further intervention within the proximal common bile duct, as follows: the taut cholangiogram catheter was removed and a laparoscopic Maryland grasper (Medline Industries, Northfield, Illinois, US) was used to thread a 0.89 mm diameter, 180 cm length, flexible tip length 3 cm angled Radifocus® Glidewire® (Terumo, Tokyo, Japan, US) into the cystic duct. Under continuous fluoroscopic visualization, the glidewire was threaded to the ampulla of Vater. Upon meeting resistance at the ampulla, a 7Fr x 45cm x 0.97 mm Flexor® Check-Flo® Introducer Set (Cook Medical, Bloomington, Illinois, US) was threaded over the glidewire. The catheter was then removed and a sturdier TEMPO AQUA® catheter (Cordis, Milpitas, California, US) was threaded into the sheath over the glidewire and passed through the ampulla of Vater. This enabled the glidewire to pass through the TEMPO AQUA® catheter (Figure 3). A cholangiogram was then repeated and demonstrated persistent constriction of the ampulla of Vater and reflux of contrast into the pancreatic duct.

**Figure 4:**
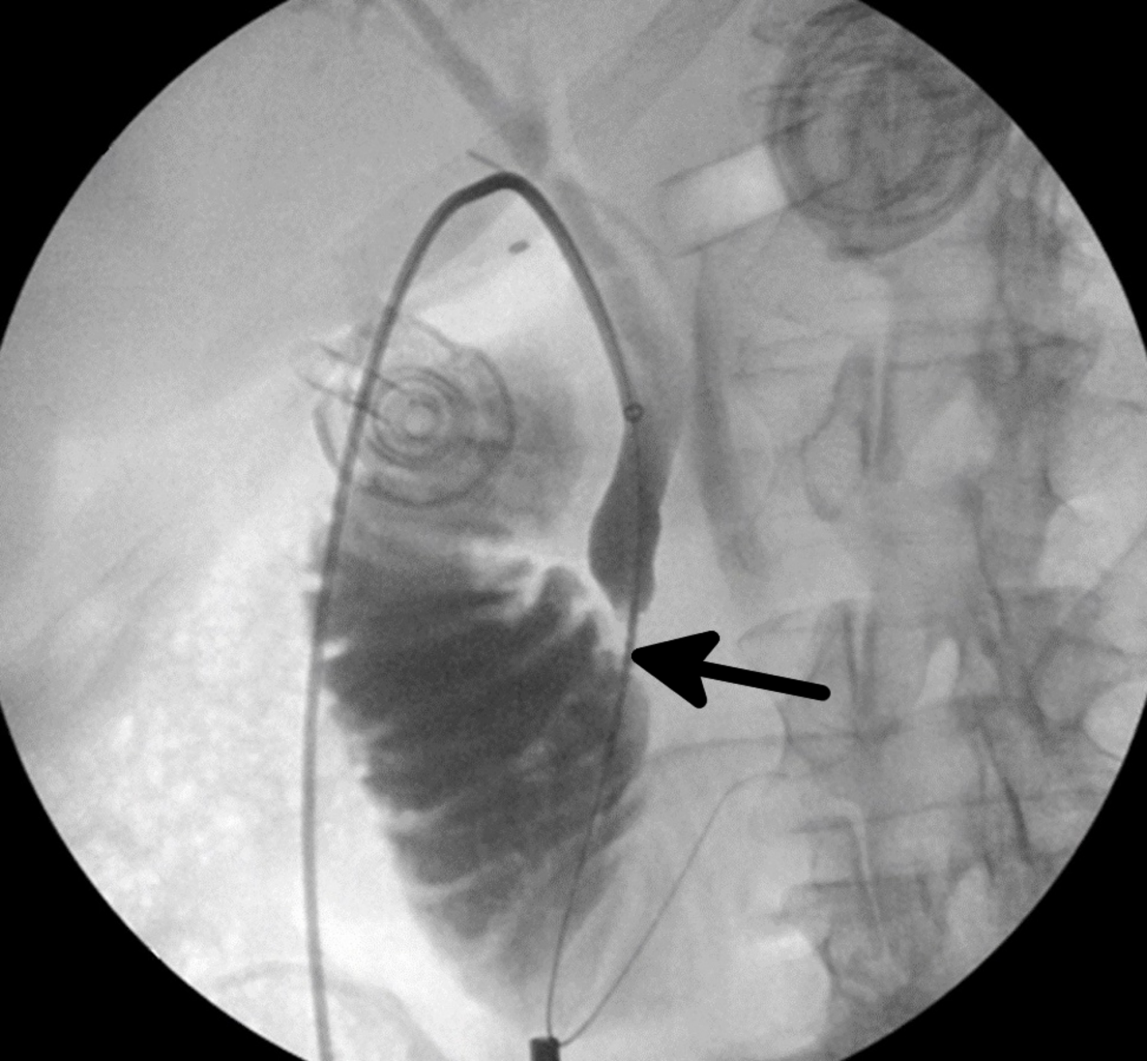
Intraoperative cholangiogram depicting moderate relief of common bile duct obstruction and flow of contrast into the duodenum following balloon dilation of the ampulla of Vater. However, the ampulla is still opacified (black arrow), suggesting possible persistence of a gallstone.

At this time, the imaging indicated the possibility of persistent occlusion with stones or a possible stricture of the ampulla. The TEMPO AQUA® catheter was then retrieved and a 4 mm x 20 mm x 75 cm Mustang Percutaneous Transluminal Angioplasty (PTA) Balloon Dilation Catheter (Boston Scientific, Marlborough, Massachusetts, US) was passed over the glidewire and through the ampulla. This balloon was then inflated and the cholangiogram was repeated, demonstrating a modest improvement in the flow of contrast through the duodenum (Figure 4). Due to the persistence of the opacification of the ampulla, the dilation was repeated with a 7 mm x 20 mm x 75 cm Mustang PTA Balloon Dilation Catheter (Boston Scientific).

**Figure 5:**
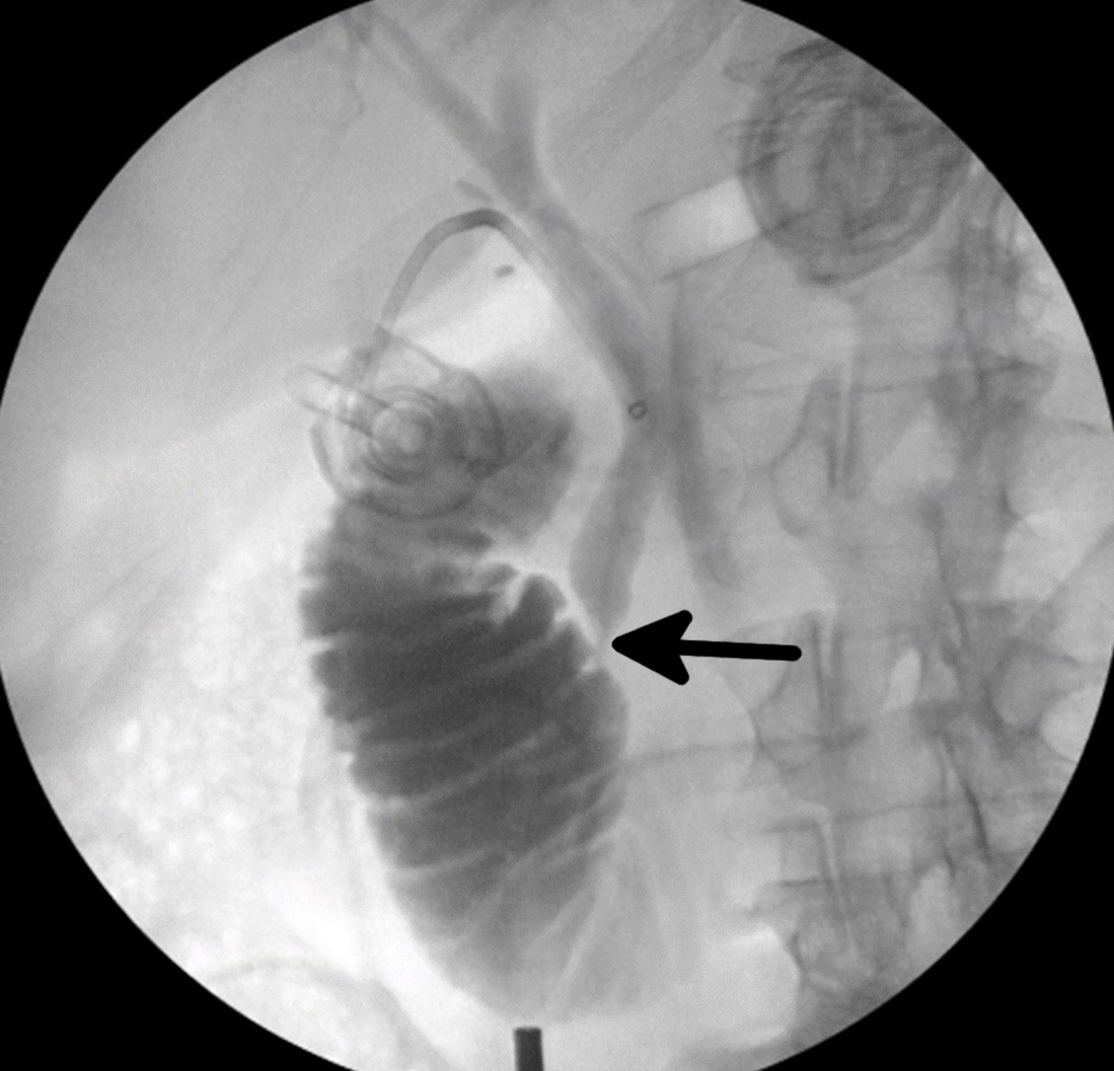
Repeat cholangiogram following a second dilation indicative of therapeutic success as there is free flow of contrast through the biliary system and through the ampulla of Vater (black arrow).

This second dilation opened up the ampulla and repeat IOC demonstrated free-flowing contrast medium into the duodenum from the cystic duct (Figure 5). The glidewire and balloon catheter and sheath were removed under direct laparoscopic guidance. The cystic duct was then transected with laparoscopic scissors and the proximal stump of the cystic duct was tied off with two PDS II ENDOLOOP® violet monofilament sutures (Ethicon, Somerville, New Jersey, US). The gallbladder was dissected off the fossa and a drain was left within the subhepatic space. The following day, the patient demonstrated a slight increase in his total bilirubin to 1.8 mg/dl but remained asymptomatic and had an unremarkable recovery following surgery. The following day, his total bilirubin decreased to 0.8 mg/dl and the patient was discharged tolerating a full diet, with minimal pain and no complications. He was discharged post-op day 1 and his subsequent follow-up appointments were uneventful for six months post-admission.

## Discussion

Many approaches have been utilized in the treatment of choledocholithiasis, including the endoscopic, laparoscopic, and open surgical techniques. Due to the delicate and variable structure of the biliary tree, the ideal management of choledocholithiasis would provide complete therapeutic efficacy and safety while remaining as minimally invasive as possible. The advantages of prior minimally invasive endoscopic techniques are currently offset by limitations in efficacy. surgeon inexperience, and suboptimal therapeutic outcomes, such as increasing the rate of converting from laparoscopic to open cholecystectomy in 15% to 27% of cases [11-12]. Occlusions or inflammations of the biliary tree require progressively more invasive maneuvers to map the biliary tree, such as laparoscopic choledochotomy or open exploration. While these invasive techniques provide higher rates of definitive treatment, they come with higher rates of complications, such as biliary tree stricture, leakage, and higher overall costs [13-14]. However, the technique discussed in this case combines the thoroughness of an open or laparoscopic common bile duct exploration without introducing any additional manipulation of the biliary tree beyond what has been done to generate the IOC. Of note, our technique of using endovascular instruments to provide clearance of the biliary tree was performed in a patient with frank gangrene of the gallbladder and diffuse inflammation of the biliary tree, which are associated with the highest risks of complications in other approaches [15]. While this case report details the technique in a patient with choledocholithiasis and cholecystitis, the same technique could be applied in patients with strictures, sludge, or stones in the common bile duct, as even small stones have been reported to increase the risk of pancreatitis [16].

There are currently no clear guidelines for the choice of trans-cystic CBD exploration versus choledochotomy for the treatment of choledocholithiasis [10]. The efficacy of the laparoscopic trans-cystic approach has been demonstrated, and it should maintain its role in many cases [10]. However, the trans-cystic approach can be limited by an unfavorable bile duct anatomy, large stone size, and a large number of stones [17-18]. If the stones are small, near the ampulla of Vater, and few in number, a traditional laparoscopic trans-cystic CBD exploration should be considered. However, if intraoperative imaging demonstrates a low likelihood of success for the traditional method, or if initial attempts to clear the stones have failed, then this technique can be used as a second line strategy to help flush such stones into the duodenum safely.

Although this procedure provided a satisfactory outcome in this instance, demonstrating the effective clearance of the common bile duct and sphincteroplasty, its long-term viability will require further evaluation with increased patient enrollment and randomization. Additionally, we acknowledge that there will be a learning curve for the mastery of this approach, as the attending surgeon who performed this procedure trained in a general surgery residency and vascular surgery fellowship. However, the time required to become competent in this technique is far shorter than the time required to become proficient at the traditional laparoscopic approach. He has been able to instruct several colleagues, who have not undergone vascular surgery fellowship training, on the use of this technique, and they have found the technique far easier and safer than their former techniques of CBD exploration.

## Conclusions

Instruments developed for the treatment of occlusive endoluminal contents in common vascular procedures can be a useful adjunct for patients with choledocholithiasis because of their durability and flexibility. The technique described in this case preserves the minimally invasive nature of laparoscopic cholecystectomy with intraoperative cholangiogram by utilizing the same incision sites. Moreover, this method is safe and offsets the potential morbidity and cost associated with subsequent ERCP that is commonly performed postoperatively for these patients. This technique may be able to maximize efficacy, safety, and cost-efficiency in the treatment of choledocholithiasis.
